# A novel missense variant in the *CASK* gene causes intellectual developmental disorder and microcephaly with pontine and cerebellar hypoplasia

**DOI:** 10.1186/s12920-022-01275-z

**Published:** 2022-06-06

**Authors:** Sixian Wu, Chuan Jiang, Jiaman Li, Guohui Zhang, Ying Shen, Jing Wang

**Affiliations:** 1 Joint Laboratory of Reproductive Medicine, Gynaecology and Paediatric Diseases and Birth Defects of Ministry of Education, West China Second University Hospital, Sichuan University, Chengdu, 610041 People’s Republic of China; 2 West China School of Pharmacy, Sichuan University, Chengdu, 610041 People’s Republic of China; 3 Department of Obstetrics and Gynaecology, West China Second University Hospital of Sichuan University and Key Laboratory of Birth Defects and Related Diseases of Women and Children, Sichuan University, Ministry of Education, Chengdu, 610041 People’s Republic of China

**Keywords:** Case report, MICPCH, *CASK*, Missense variant, Protein structure

## Abstract

**Background:**

Variants in the *CASK* gene result in a wide range of observed phenotypes in humans, such as FG Syndrome 4 and intellectual disabilities. Intellectual developmental disorder with microcephaly and pontine and cerebellar hypoplasia (MICPCH) is an X-linked disorder that affects females and is characterized by severely impaired intellectual development and variable degrees of pontocerebellar hypoplasia. Variants in *CASK* are the main genetic cause of MICPCH. Variants in *CASK* can explain most patients with MICPCH, but there are still some patients whose disease aetiology cannot be explained.

**Case presentation:**

An 11-month-old female diagnosed with MICPCH exhibited general developmental delays, microcephaly, and cerebellar hypoplasia. Whole-exome sequencing (WES) was used to find a novel heterozygous missense variant (NM_003688.3: c.638T>G) of *CASK* in this patient. Strikingly, this variant reduced the expression of CASK at the protein level but not at the mRNA level. By using protein structure prediction analysis, this study found that the amino acid change caused by the variant resulted in further changes in the stability of the protein structure, and these changes caused the downregulation of protein expression and loss of protein function.

**Conclusion:**

In this study, we first reported a novel heterozygous pathogenic variant and a causative mechanism of MICPCH. The amino acid change cause by this variant led to changes in the protein structure and a decrease in its stability, which caused a loss of protein function. This study could be helpful to the genetic diagnosis of this disease.

**Supplementary Information:**

The online version contains supplementary material available at 10.1186/s12920-022-01275-z.

## Background

Intellectual developmental disorder and microcephaly with pontine and cerebellar hypoplasia (MICPCH) is an X-linked disorder that affects females and is characterized by severe intellectual disability, microcephaly, and variable degrees of pontocerebellar hypoplasia [1, 2]. Affected individuals have very poor psychomotor development, often without independent ambulation or speech, and axial hypotonia with or without hypertonia. Some affected individuals may have sensorineural hearing loss or eye abnormalities. The dysmorphic features of those affected by this condition include overall poor growth and severe microcephaly (− 3.5 to − 10 SD). This causes the development of distinct facial features, such as broad nasal bridge and tip, large ears, long philtrum, micrognathia, and hypertelorism [3, 4]. It has been reported that variants of *CASK*, *ITPR1*, *MARCKS*, and *RELN* are involved in the aetiology of MICPCH [5, 6]. *CASK* is an excellent candidate gene for microcephaly disproportionate pontine and cerebellar hypoplasia (MICPCH) (MIM# 300749) since this gene functions in neuronal development. *CASK* mutant mice have small brains, abnormal cranial shapes, and cleft palates [4, 6–10]. Intragenic variants of *CASK* have been found in more than 50 individuals with the MICPCH phenotype [5, 11].

The *CASK* gene is a member of the MAGUK protein family. It maps to Xp11.4 and encodes CASK. CASK is a multidomain scaffolding protein composed of 926 amino acids (Ensembl ID: ENST00000378163) that is located at both the postsynaptic membrane of central nervous synapses and within the nuclei of neurons. *CASK* is highly expressed in the foetal brain [8, 12, 13]. Due to its location on the X chromosome, the loss-of-function of *CASK* usually leads to the manifestation of MICPCH in females [3, 5]. Males affected by *CASK* variants usually show more severe symptoms than females. These genetic issues are usually fatal in the womb for male embryos [7, 14].

In the clinical screening for this study, we first examined the case of an 11-month-old female patient with general developmental delay, microcephaly, and cerebellar hypoplasia. Whole exome screening indicated that the patient had a novel heterozygous missense variant in the *CASK* gene at the location NM_003688.3: c.638T>G, p.L213R. In this study, we used bioinformatics methods to predict the pathogenicity and harm of this missense variant in *CASK*. We also examined the effects of the variant on the mRNA and protein expression of *CASK* and predicted the structure of the protein. This study found that there is no difference in mRNA expression levels. However, amino acid variants were found to cause downstream changes in the stability of the spatial structure of the protein, and these changes downregulate protein expression and cause a loss of protein function.

## Case presentation

### Clinical summary

**Fig. 1:**
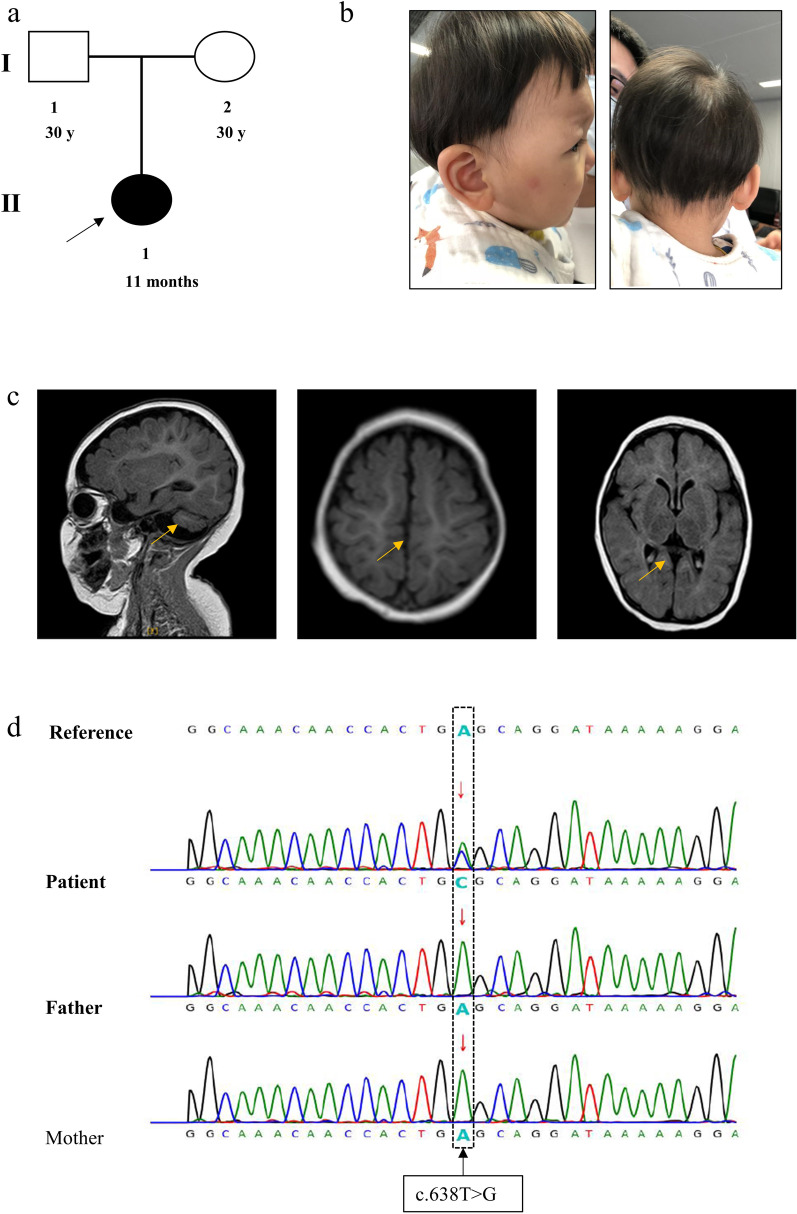
Clinical summary for the patient. **a** Family pedigree. An unrelated natural couple who gave birth to the affected female. The black arrow denotes the proband. **b** The patient’s figure. **c** The patient’s axial brain MRI, which indicates the widening of the cerebral fissure and the shrinkage of the cerebellum. **d** PCR sequencing confirmed the *CASK*: NM_003688.3: exon 7: c.638T>G: p. L213R mutation in this family

An unrelated natural couple brought an 11-month-old female with delayed development to the outpatient department for genetic counselling (Fig. 1a). There were no obvious abnormalities detected during the foetal period for this patient. After birth, the baby was found to have jaundice (lasting for 1 month), difficulties falling asleep and a small head circumference. At the age of 7 months, physical examination showed muscular hypertonia, hands often clenched, and global growth regression. The electroencephalogram showed bounded linearity. Cranial MRI showed that the volume of the bilateral cerebellar hemispheres was significantly decreased, especially in the lower part of cerebellar hemisphere, the cerebellar sulcus was widened and deepened, and the occipital cistern was widened (suspected cerebellar hemisphere dysplasia). Additionally, the signal of the bilateral globus pallidus changed slightly, the sulci of the bilateral cerebral hemispheres was slightly widened and deepened, the bilateral frontotemporal extracerebral space was slightly widened, and the bilateral lateral ventricles were slightly widened. At 11 months of age, the head circumference was 40 cm (< − 3 SD) (reference value: 41.9–47.3 cm) (Fig. 1b). The clinical diagnosis was primary microcephaly. There was no genetic history in the family, and the parents were not close relatives (Fig. 1c). We initially diagnosed the patient with intellectual developmental and microcephaly with pontine and cerebellar hypoplasia (MICPCH) based on the specific clinical characteristics. There were no phenotypic abnormalities observed in the parents of the patient.

### Molecular genetic analysis

We extracted DNA from the patient’s peripheral blood sample and performed WES. The results showed that the *CASK* gene had a heterozygous missense variant, specifically *CASK*: NM_003688.3: exon 7: c.638T>G: p.L213R. (Fig. 1d) [15].

**Fig. 2:**
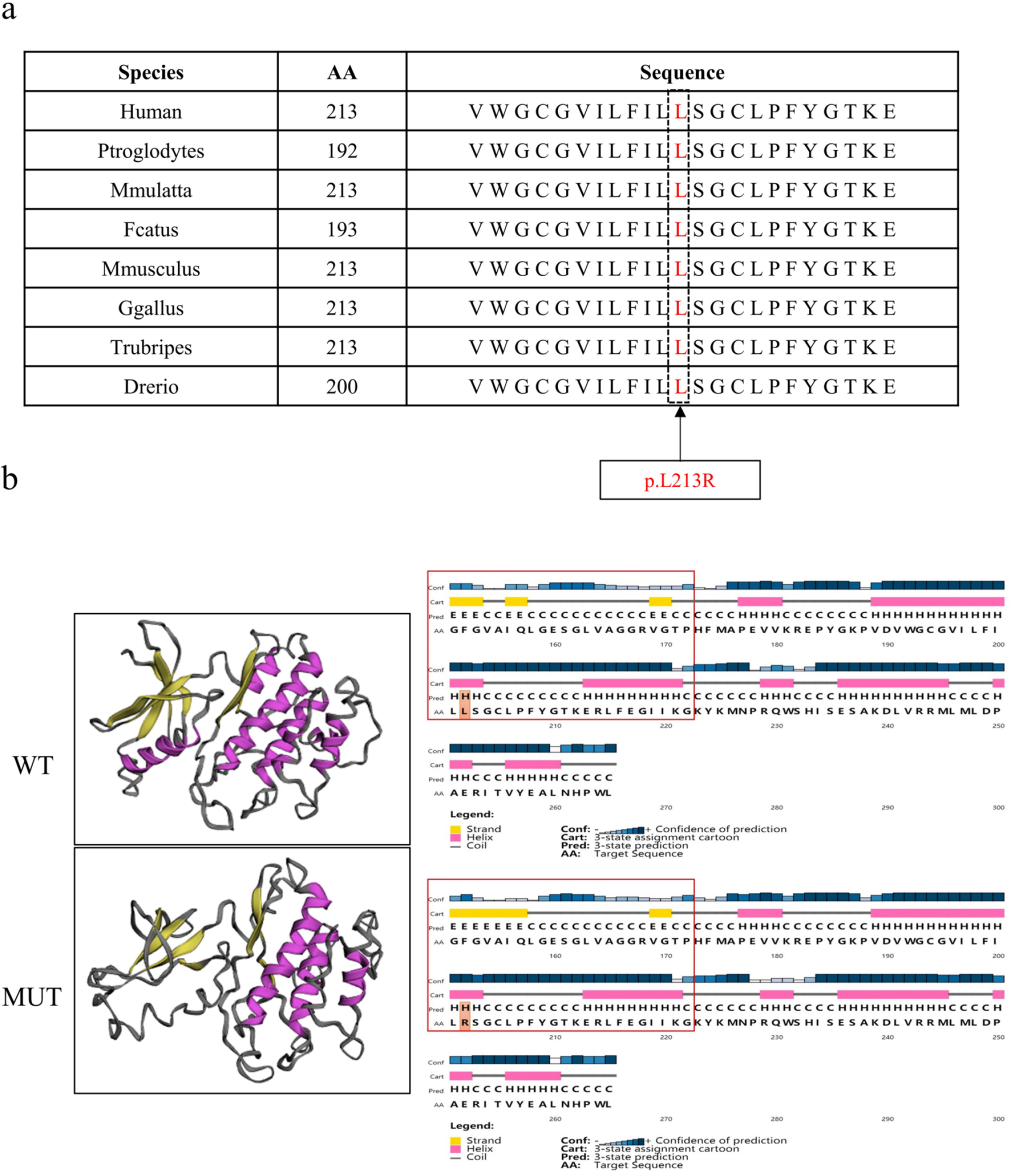
Bioinformatic analysis of the conservation and pathogenicity of the variant in *CASK*. **a** Multiple sequence alignment of the CASK protein for different species. The black arrow denotes the position of the variant (c.638T>G: p.L213R). **b** The secondary and spatial structure prediction of the WT and MUT proteins

According to the bioinformatics analysis, this variant was not previous reported and was not found in most databases, including the ExAC browser, 1000 Genomes Project, and In-house Chinese-Control. The latest gnomAD database indicates that the frequency of this variant is 0.000005520 [16] (Table 1). In addition, this variant site is highly conserved in many species according to mutation taster (Fig. 2a). PhastCons and PhyloP were used to evaluate the scores of amino acid sequence conservation. The scores indicated that this variant site is highly conserved (Table 1). Moreover, this mutation was predicted to be deleterious by the following bioinformatic tools: SIFT [17], PolyPhen-2 [18], and M-CAP [19] (Table 1). The above results indicate that this variant site is pathogenic and well conserved.

**Table 1:** Information on missense mutations in the *CASK* gene

Gene	CASK	
cDNA mutation	NM_003688.3: c.638T>G	
Variant allele frequency	ExAC Browser	0
	GnomAD	0.000005520
	1000 Genomes Project	0
	In-house Chinese-Control	0
Amino acid sequence conservation (mutation taster)	PhyloP	3.907
	PhastCons	1
Function prediction	SIFT	Deleterious
	PolyPhen-2	Most likely, damaging
	M-CAP	Possibly pathogenic

### Pathogenicity analysis of the CASK variants

**Fig. 3:**
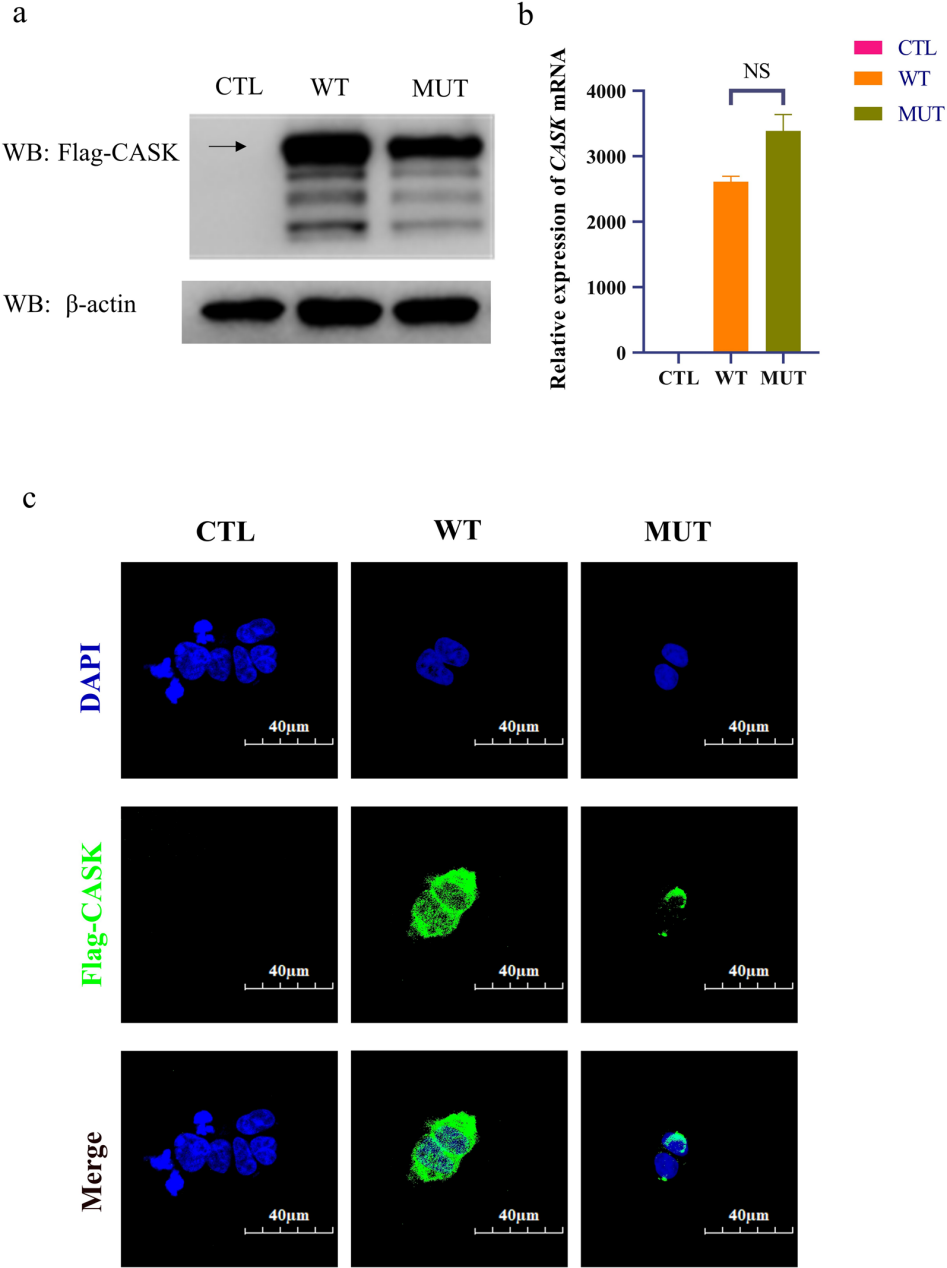
The negative effect of this variant on *CASK* expression. **a** Western blotting revealed a downregulation in the expression of the CASK protein after mutation. **b** qPCR revealed that there was no significant difference in the mRNA expression of *CASK* between the WT and MUT. **c** Immunofluorescence revealed a downregulation in the expression of the CASK protein after mutation

To further confirm the negative effect of this variant on *CASK* expression, wild-type and mutant plasmids were constructed and transfected into HEK-293T cells. We determined the mRNA (Fig. 3b) and protein expression (Fig. 3a, c) of both the wild type and the mutant and found that there was no significant difference in mRNA expression between the wild type and the mutant. However, compared with the wild type, the protein expression of the mutant was downregulated.

Finally, we predicted the structural pattern of the protein after the amino acid arginine (R) was substituted for leucine (L) by PSIPRED [20–22]. Importantly, the mutant protein showed decreased protein stability, which is represented by the increased Gibbs free energy (ΔΔG_pred_ = 1.857). The results of the protein structure prediction showed that the nuclear charge of the protein increased (ΔCharge = 1) and the stability of the protein decreased (ΔΔG_pred_ = 1.857) after the variant. Moreover, a random coil in the secondary structure is changed to a β-sheet, which also affects its spatial structure (Fig. 2b). Therefore, the decreased protein stability and the changed protein structure might contribute to the downregulation of CASK protein expression, further causing the loss of protein function.

## Discussion and conclusions

In this study, we examined the case of a female with a novel heterozygous pathogenic missense variant in *CASK* that is associated with MICPCH. Moreover, the MRI results revealed a decreased size in both cerebellar hemispheres, a widening of the sulci in both cerebral hemispheres, and a widening and deepening of the cerebellar sulci. The WES results showed that the *CASK* gene had a heterozygous missense variant: NM_003688.3: exon 7: c.638T>G: p.L213R. Although there was no significant difference in the expression of *CASK* at the mRNA level between the wild type and mutant genes, the protein expression of CASK was downregulated. By using the protein structure prediction method, we found that when leucine was mutated to arginine in the primary sequence of the protein, the protein stability was reduced. Additionally, the secondary structure and spatial structure were changed. This resulted in reduced protein expression and the loss of protein function.

All residues in the αF helix have been shown to be involved in a conserved spatial pattern. Kornev et al. [23] suggested that the residue at the position equivalent to 209 in CASK plays a critical role in the anchoring of the αH helix, which is purported to help stabilize the hydrophobic core around the αF helix [12]. Similarly, in this study, the leucine at position 213 in the amino acid sequence of the CASK protein was mutated to arginine. Arginine is a basic amino acid and leucine is a nonpolar amino acid [24]. Thus, this amino acid change alters the tertiary structure of the protein [25], which probably impedes the anchoring of the αH helix. Furthermore, the destabilizing αH helix may not stabilize the hydrophobic core around αF, which may greatly disrupt the function of *CASK* as a protein kinase.

Generally, the types of variants that cause MICPCH are nonsense mutations, splice site mutations, frameshift mutations, and missense mutations. These types of variants usually result in a decrease or absence in CASK protein expression. The loss of *CASK* expression is associated with a more severe MICPCH phenotype and likely causes reduced viability or in utero lethality [7]. Missense variants in the *CASK* gene result in a mild intellectual developmental disorder that sometimes includes nystagmus, most often in males [26]. Our study also reports a missense variant, but the phenotype caused by this missense variant is more severe than that reported in a previous patient with a missense variant in *CASK*. This might be due to differences in the genetic backgrounds. Moreover, we found that this missense variant could change the properties of the amino acid sequence and changes the protein structure and stability of the CASK protein. These changes may lead to the downregulation of protein expression, further causing the loss of protein function.

In conclusion, this study reports a novel heterozygous pathogenic missense variant in *CASK*, which expands the spectrum of genetic alterations that cause *CASK* mutations. Moreover, this research is of great significance for understanding the pathogenicity of *CASK* point variants. This study further reveals the key role of *CASK* in MICPCH development and helps provide genetic explanations for the aetiology of *CASK* variants in MICPCH-affected patients.

## Supplementary Information

**Additional file 1.** Methods and Materials in this study.

## Data Availability

All data generated or analysed in this study are included in this published article and its Additional file 1. The raw datasets used and analysed during the current study are not deposited in publicly available repositories because of considerations about the security of human genetic resources. For other details of the availability of data and material, please refer to the methods section of the article and tables. Sequencing dataset can be obtained from the corresponding author on reasonable request.

## Abbreviations

MICPCH: Intellectual developmental disorder and microcephaly with pontine and cerebellar hypoplasia
FGS4: FG Syndrome 4
MRI: Magnetic resonance imaging
WES: Whole-exome sequencing
