# Supplementary material for: A novel missense variant in the CASK gene causes intellectual developmental disorder and microcephaly with pontine and cerebellar hypoplasia
Source: BMC Med Genomics. 2022 Jun 6;15:127. doi: 10.1186/s12920-022-01275-z (PMC9169347; doi:10.1186/s12920-022-01275-z)
Supplement: Supplementary file 1 — Additional file 1. Methods and Materials in this study. [file 12920_2022_1275_MOESM1_ESM.docx]

**A novel missense variant in the *CASK* gene causes intellectual developmental disorder and microcephaly with pontine and cerebellar hypoplasia**

Sixian Wu ^1,2,$^, Chuan Jiang ^1,$^, Jiaman Li ^2^, Guohui Zhang ^1^, Ying Shen^1,#^ and Jing Wang^3,#^

$Equal contribution: Sixian Wu and Chuan Jiang should both be regarded as first authors.

#Corresponding author:

Ying Shen, Email: [yingcaishen01@163.com](mailto:yingcaishen01@163.com); or. Jing Wang, Email: [hhwj_123@163.com](mailto:hhwj_123@163.com)

^1^ Joint Laboratory of Reproductive Medicine, Gynaecology and Paediatric Diseases and Birth Defects of Ministry of Education, West China Second University Hospital, Sichuan University, Chengdu 610041, P. R. China.

^2.^ West China School of Pharmacy, Sichuan University, Chengdu 610041, China.

^3^. Department of Obstetrics and Gynaecology, West China Second University Hospital of Sichuan University and Key Laboratory of Birth Defects and Related Diseases of Women and Children, Sichuan University, Ministry of Education, Chengdu 610041, China.

**Methods**

*Subjects*

Peripheral blood samples were obtained from the proband and her family after informed consent was signed by the patient and all family members. This experiment on human subjects was approved by the Ethical Review Board of West China Second University Hospital, Sichuan University. The patient’s brain MRI results were evaluated by the pediatric neuroradiologist.

*Whole-exome sequencing*

Genomic DNA was extracted from peripheral blood leukocytes using a whole blood DNA purification kit (TIANGENE). For WES, exons were captured from 1 μg genomic DNA using high-throughput sequencing detection technology for the whole exome provided by the manufacturer. The Verita Trekker® variant site detection system and Enliven® variant site annotation interpretation system independently developed by Berry Genomics were used to analyze the data. Functional annotation was performed through ANNOVAR, and data were filtered by public databases such as ExAC, 1000 Genomes Project, and GnomAD .

Candidate pathogenic variants in the family members and their flanking intronic regions of *CASK* in the unrelated population were validated by Sanger sequencing. PCR amplification was performed with Dyad Polymerase (Bio-Rad Laboratories). DNA sequencing of PCR products was conducted on an ABI377A DNA sequencer (Applied Biosystems). The primers for PCR are listed as follows:

5′ TTTCCTGGACAGCTACAGGC 3′, 5′ CTGGCAGTCAATTAGTGGGCA 3′

*Plasmid construction and cell transfection*

The full-length cDNA of *CASK* was synthesized and separately cloned into pCMV-MCS-3*Flag. The *CASK* plasmids were synthesized and cloned by Gene Company (Shanghai, China). All mutant plasmids of *CASK* were generated by the Fast Mutagenesis System of TransGen Biotech Co. Ltd (Beijing, China). The constructed plasmid was transformed into *E. coli*. Then, 10 μL of plasmid-containing bacterial solution and 20 μL of antibiotic kanamycin (50 mg/mL) were added to 20 mL of Luria-Bertani medium. The bacteria were placed on a shaker at 37°C overnight. After shaking was complete, researchers extracted the plasmid from *E. coli*.

The HEK-293T cell line was obtained from the American type culture collection (ATCC, USA). The 293T cells were cultured in 6-well cell culture plates and 100 mm cell culture dishes (WHB, China) with basic DMEM medium containing 10% fetal bovine serum (Gibco, USA) and 0.1% penicillin/streptomycin in a humidified incubator at 37°C with 5% CO_2_. According to the experimental scheme, *CASK* plasmids were transfected into 293T cells for 24–48 h.

*Quantitative PCR*

The total RNA of the cell was extracted using TRIzol reagent (Invitrogen) and was converted to cDNA using a Revert Aid First-Strand cDNA Synthesis Kit (ThermoFisher). Quantitative PCR was performed using SYBR Premix Ex Taq II (TaKaRa) on an iCycler RT-PCR Detection System (Bio-Rad Laboratories).

The ΔΔCT method was used for data analysis. Each assay was performed in triplicate for each sample. The *GADPH* gene was used as an internal control.

The primers of human *GADPH* are as follows:

5′ ATGTTCGTCATGGGTGTGAA 3′,

5′ GTCTTCTGGGTGGCAGTGAT 3′

The primers of human *CASK* are as follows:

5′ GACTTGTAGCTGGAGGACGTG 3′

5′ GATATGGCTCCACTGCCTTGG 3′

*Western blotting*

The proteins were extracted using radioimmunoprecipitation assay (RIPA) buffer that contained a protease and phosphatase inhibitor cocktail (Roche). Twenty microgram of the denatured proteins were separated with the use of 10% SDS-polyacrylamide gels and transferred to a polyvinylidene difluoride (PVDF) membrane (Millipore) for immunoblotting analysis. After blocking with Tris-buffered saline/Tween-20 (TBST) containing 5% bovine serum albumin (BSA) for 1 h at room temperature, the membranes were then incubated with the corresponding primary antibodies 1:50 anti-FLAG (HPA052219; Sigma-Aldrich) and 1:2000 anti-β-actin (T7451; Sigma-Aldrich) at 4°C. Samples were incubated overnight. The binding of the primary antibodies was visualized using horseradish peroxidase-conjugated goat antirabbit or antimouse IgG (1:10,000, ZSGB-BIO, China). The signal intensities were measured using ECL (1305702; Millipore Corporation, Billerica, USA) and image analysis software (ImageJ, NIH).

*Immunofluorescence*

The cells transferred into the plasmid were cultured in a glass-bottom dish. Then the cell samples were fixed onto slides with using 4% paraformaldehyde for 10 min, the followed by slides were washed by with PBS. The slides were permeabilisedzed with 0.3% Triton X-100 and blocked with 5% BSA in PBS. Next, the slides were then incubated with the corresponding primary antibodies (anti-FLAG 1:50) overnight at 4 °C. Second on the following day, the slides were washed by PBS, then incubated with Alexa Fluor 488 (1:500) (A21206; Thermo Fisher) labelled secondary antibodies for 1h at room temperature, and then counterstained with 4,6-diamidino-2-phenylindole (DAPI,) (Sigma-Aldrich) to label the nuclei. Images were obtained using a laser scanning confocal microscope, 60X (Olympus).
